# The unique probe selector: a comprehensive web service for probe design and oligonucleotide arrays

**DOI:** 10.1186/1471-2105-9-S1-S8

**Published:** 2008-02-13

**Authors:** Shu-Hwa Chen, Chen-Zen Lo, Ming-Chi Tsai, Chao A Hsiung, Chung-Yen Lin

**Affiliations:** 1 Stem Cell/Regenerative Medicine Program, Genomics Research Center, Academia Sinica., No. 128 Academia Rd., Sec. 2, Taipei 115, Taiwan; 2 Institute of Information Science, Academia Sinica, No. 128 Academia Rd., Sec. 2, Taipei 115, Taiwan; 3 Division of Biostatistics and Bioinformatics, National Health Research Institutes. No. 35 Keyan Rd. Zhunan, Miaoli County 350, Taiwan; 4 Institute of Fishery Science, College of Life Science, National Taiwan University, No. 1, Roosevelt Rd. Sec 4, Taipei, Taiwan

## Abstract

**Background:**

Nucleic acid hybridization, a fundamental technique in molecular biology, can be modified into very effective and sensitive methods for detecting particular targets mixed with millions of non-target sequences. Therefore, avoiding cross-hybridization is the most crucial issue for developing diagnostic methods based on hybridization.

**Results:**

To develop a probe with a high discriminating power, this study constructed a web service, the Unique Probe Selector (UPS), for customized probe design. The UPS service integrates a probe design mechanism and a scoring system for evaluating the performance of probe annealing and the uniqueness of a probe in a user-defined genetic background. Starting from an intuitive web interface, the UPS accepts a query with single or multiple sequences in fasta format. The best probe(s) for each sequence can be downloaded from result pages in a fasta or .csv format with a summary of probe characteristics. The option "***Unique probe within group***" selects the most unique probe for each target sequence with low probability to hybridize to the other sequences in the same submitted query. The option "***Unique probe in the specific organism***" devises probes for each submitted sequence to identify its target among selected genetic backgrounds based on Unigene.

**Conclusion:**

The UPS evaluates probe-to-target hybridization under a user-defined condition *in silico *to ensure high-performance hybridization and minimizes the possibility of non-specific reactions. UPS has been applied to design human arrays for gene expression studies and to develop several small arrays of gene families that were inferred as molecular signatures of cancer typing/staging or pathogen signatures. Notably, UPS is freely accessible at .

## Background

Nucleic acid hybridization is one of the most fundamental techniques in molecular biology. The universal role of nucleic acid in transmitting genetic information and the characteristics of these molecules to form complementary duplexes make this technique widely applicable in various biological and medical fields. Under an optimal protocol, nucleic acid hybridization can be modified into very effective and sensitive methods for detecting target molecules. For example, a typical polymerase chain reaction (PCR) [1] can amplify a fragment of a particular target DNA template even when only one target molecule is mixed in with millions of non-target sequences. The DNA microarray [2] is another good application of nucleic acid hybridization technology. This method has become increasingly popular in biological and medical research due to its availability and capability of inspecting whole-gene expression profiles simultaneously in a high-throughput manner. The specificity of these widely applied methods depends on the primer (or probe)-to-target hybridization; cross-hybridized signals can lead researchers to make incorrect conclusions.

Avoiding cross hybridization is an important issue when developing assays based on hybridization. Some general considerations exist for oligonucleotide probe design, such as probe length, GC content, stability of probe-target duplex, and probe folding. These parameters are difficult to calculate without computational tools. Furthermore, hybridization experiments conducted in a wet-lab bench are typically utilized to detect target(s) from many different molecules chaotically mixed together. Guaranteeing that a probe picked from its target sequence will not stick to other molecules in the reaction mixture is difficult. Fortunately, as the sequence database content has increased, evaluating the hybridization background of a probe in a given genetic background is possible. For instance, blastn can be employed to check for the existence of highly complementary sequences other than the target, which can form binding pairs in the reaction.

Several tools are available for probe design. Most of these are performed in command mode, and only few provide a Graphic User Interface (GUI). For example, OligoArray 2.1 [3,4], GoArrays [5], OligoPicker [6], ArrayOligoSelector [7] and Picky [8] are stand-alone application tools run in command mode and provide unique probes for input sequences under the constrains of GC%, Tm, absence of low complexity and position near the 3'end. OligoWiz2 [9] is a java-base server-client solution for probe design in a graphical user interface. Oligodb [10] is a database of pre-calculated probes for human and mouse transcriptome. Although most of these tools are utilized for designing probes for DNA microarrays, few consider genetic background noise in hybridization reactions. A web-based tool for designing probes that can select probes for targets with small likelihood of cross-hybridization is sparse.

This study presents a novel web tool, the Unique Probe Selector (UPS), that can select unique oligonucleotide probes. The algorithms applied in this study include thermodynamic theory, GC content, GC clamps, secondary structure of probes and some other empirical preferences of wet-lab researchers. Low-complexity regions are filtered out by the mechanism developed in this study to maintain probe specificity. Some strategies in the UPS are similar to those in a previous primer design tool, Primer Design Assistant (PDA) [11], which has several diagnostic kits for detecting specific pathogens [12]. The UPS provides a succinct user interface for job submission. Ranging from a single sequence query to a batch-wise multiple sequences query, the UPS is a robust tool that generates probe candidates for target sequences. The UPS is the first probe design tool that can identify unique fragments in each submitted sequence in a set of query sequences, or a unique fragment in each sequence in contrast to a user-defined genetic background. The proposed tool mimics the environment of a target/non-target mixture in a true experiment and suggests a unique probe set from a candidate list, which is a very useful property in developing a diagnostic kit.

## Results

**Figure 1:**
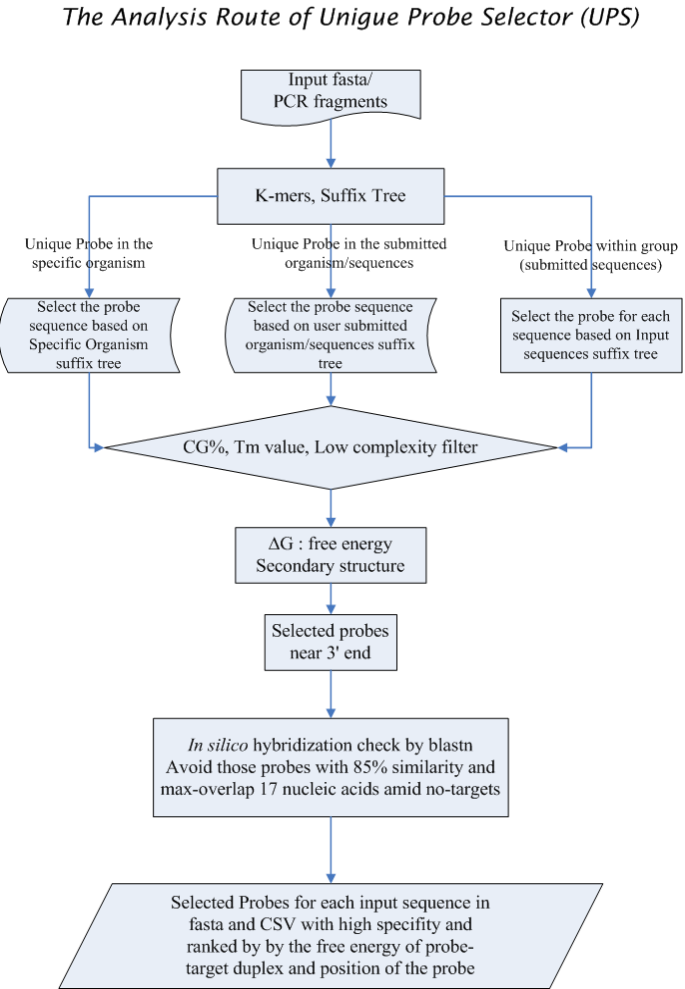
The workflow of Unique Probe Selector (UPS).

**Figure 2:**
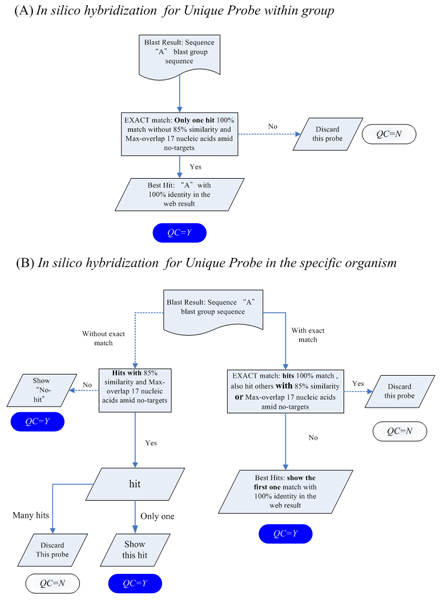
The flowchart of *in silico *hybridization. Two options, "Unique Probe within group" and "Unique Probe in the specific organism" are used to define the non-target background dataset for selecting suitable probes with low chance of cross-hybridization.

The UPS web service was developed for customized probe design and to ensure that a probe has a high discriminatory power between target and non-target sequence. The UPS combines a probe design mechanism and a scoring system for assessing the performance of probe annealing and the uniqueness of a probe in a user-defined genetic background. Figure 1 presents the UPS workflow for each query sequence string, a temporary database of substrings in a length of a user-defined 'probe length' is generated. After filtrating through the limits set in *K*-mer (*K *= 5 in this study) and in the suffix tree, survived probes are filtered again using a preset criteria, including the GC content (GC%), melting temperature (Tm) and sequence complexity, to eliminate probes with low discrimination power in hybridization. Free energy (ΔG) and the secondary structure of these probe candidates are derived to provide a score for probe-target duplex stability. Probes meeting all criteria are subjected to *in silico *hybridization. Two uniqueness options of, ***Unique Probe within a group ***and ***Unique Probe in the specific organism ***(Fig. 2, and described below) are utilized to define non-target sequences in the *in silico *hybridization test. Any probe with 85% similarity to non-target sequences or with a continuous aligned segment exceeding 17-mer in length to non-target sequences is discarded. Finally, probe candidates for each query sequence are sorted by a ranking mechanism that considers the stability of the probe-target duplex and probe positions (probes near the 3' end are favored). A flag of the final quality check on sequence uniqueness (Yes or No) is also included. For instance, if a probe of a given sequence passes all criteria but hits two Unigene sequences, it is marked by an "N," to declare for the failure of passing the final quality check.

**Figure 3:**
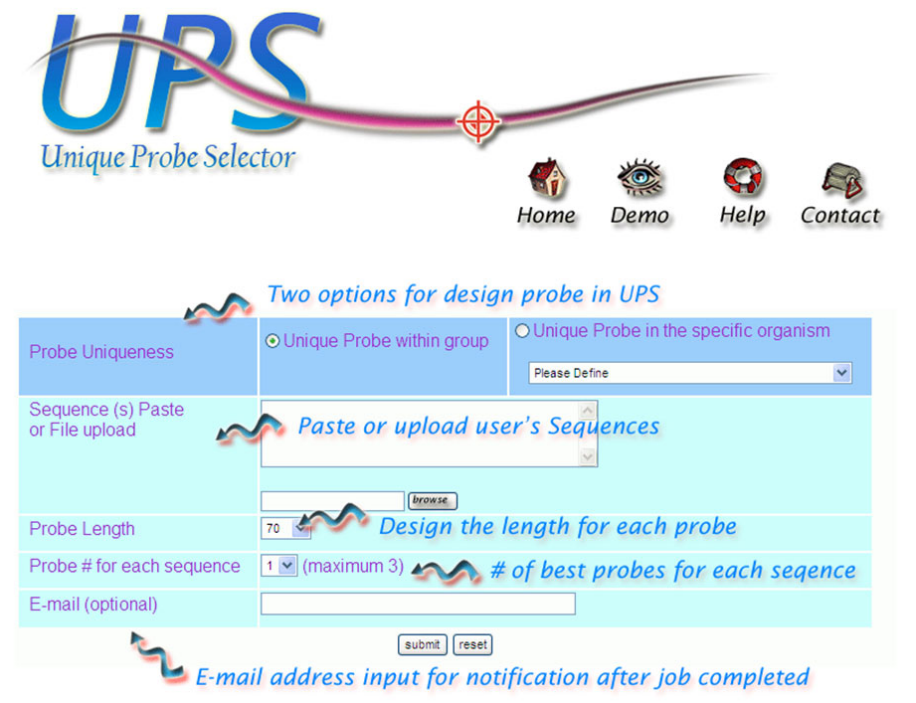
The screenshot of UPS web interface.

Probe design query on the UPS starts with a succinct and intuitive web interface (Fig. 3). Users can use various web browsers and paste nucleotide sequences in the standard fasta format directly into the submission form, or upload a plan text file containing target sequences. Upon job submission, the UPS will check input sequence for any inaccuracies, such as using a sequence identifier more than once, incorrect fasta format, non-nucleotide abbreviates, unacceptable symbols in the query, and conflict between probe length defined by a user and the submitted sequences. If any error is found, the UPS will present a warning message telling users to check their input sequences.

Two options of probe uniqueness are available. In "***Unique Probe within group***", the UPS finds the unique probe for each sequence with a low possibility to form a stable duplex with other non-target sequences in the same submitted job. In "***Unique Probe in the specific organism***," the unique probe for each sequence is not only unique among all submitted sequences but also among all available transcripts of the species chosen. To date (June 2007), 77 species are listed in UPS organism menu. This species list and completeness of the transcriptome of each species are based on the Unigene database, which is updated routinely. Generally, a batch-wise submission of 150 sequences (1 k in length on average) in the options "Unique Probe within group" and "Unique Probe in the specific organism" will take 120 and 180 sec to process, respectively. Probe design jobs for *S. pombe *whole transcriptome (≈5,000 coding sequences from Sanger center, ; probe length in 50 mers) and for *H. sapiens *coding sequences (≈17550 coding sequences from CCDS build 36.2, NCBI; probe length in 70 mers) are completed in 120 min and 4 hr in the option "Unique Probe within group", respectively. For those organisms listed in Unigene, a link is provided to retrieve pre-calculated unique probe results for each transcript in the option "Unique Probe within group". Although UPS can work on the entire transcriptome of a species, any query file >40 Mb is rejected automatically for the reason of system performance. Such a dataset may be forwarded to the authors for a solution.

**Figure 4:**
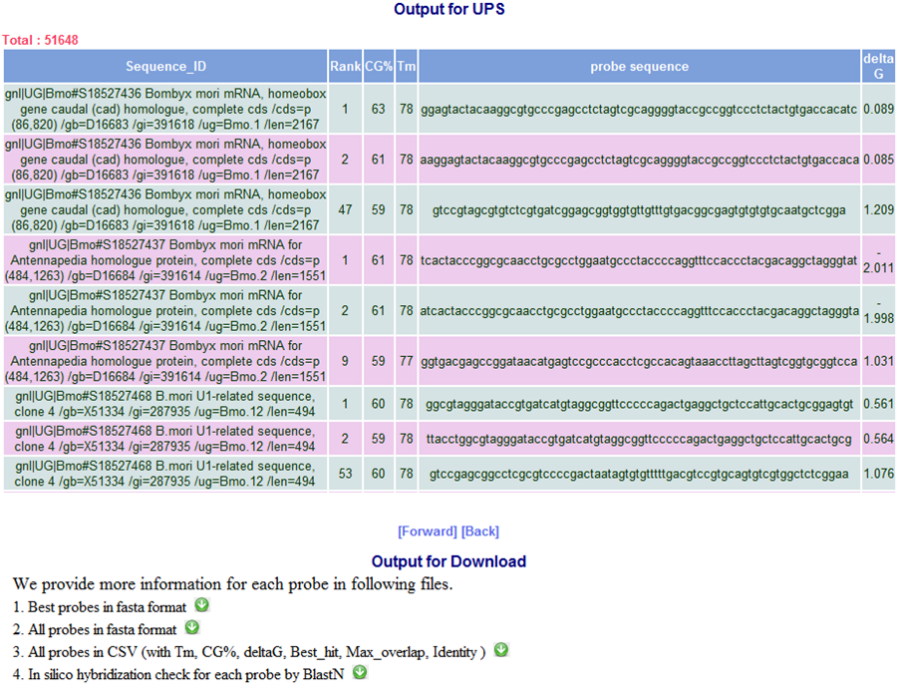
Full report of the probes designed by UPS.

For efficient system performance, the UPS can process five queries at the same time to accelerate completion time for queued jobs. However, the UPS may not response to a probe design query immediately as time may be required to complete calculations or the system may be processing several submitted jobs simultaneously. We suggest users to use the link on the submission page as a bookmark, or follow this link in the UPS notification e-mail (when an e-mail address is submitted along with the query) to revisit the result page after job completion. The best probe validated by the UPS for each sequence can be downloaded from the result page in the fasta format, as a plain text file or in a tab delimited text file. The full report, containing parameters such as Tm value and blast alignment, and a judgment of probe uniqueness by UPS, is available (Fig. 4) on the result page.

## Discussion

At time of submission, the UPS was running for over 12 months to test its stability and refine the system. Over 3000 input sequences were sent by the development team and collaborating researchers. All probes selected by the UPS satisfied several criteria, including suitable range for GC content and melting temperature, distance to the 3' end, avoiding a low complexity region, and probe-target duplex stability evaluated in theromodynamic model. For specificity, "*in silico *hybridization" based on blastn was used to ensure good performance of each probe. The species list and transcript dataset for each species, parsed from the Unigene database, is updated regularly.

Several arrays, such as whole transcriptome oligo-microarrays and small arrays for special research purposes, were designed by the UPS and have been used in wet laboratories. For instance, protein tyrosine phosphatase (PTP)/protein tyrosine kinase (PTK) families are two gene families with important roles in cell signaling pathways. How these genes are controlled in the expression level is an important question in cancer biology. Aided by the UPS, a small array for detecting members of the PTP/PTK families was designed. Preliminary array results are consistent with PCR results (manuscript in preparation). In another case, a set of unique probes for detecting pathogens commonly found as sources of biological contaminants in seafood were constructed based on the UPS. Probes of human 44 K genes were also designed by UPS, and a batch of microarray slides through the contract manufacturing service Agilent was constructed. The UPS is quite suitable and allows researchers to design probes for customized small arrays or for whole genome microarrays of species without a commercialized array platform.

The UPS is the first probe design tool for identifying unique fragments in each submitted sequence in a set of sequences in a query, or the unique fragment of each sequence in a user-defined genetic background. With a flexible and intuitive interface, users can customize their probe design following online instructions to obtain the probes with quality information. A single probe designed in the UPS can be utilized for detecting a target in conventional hybridization-based assay, such as northern blotting. Via the batch-wise query mode, unique probes selected by the UPS can meet the criteria for microarray probe design. Additionally, we are also working on creating a mechanism to admit user uploading sequences of a reference organism not listed in the current Unigene dataset. Computational method used in the UPS is a mimic of a hybridization of target/non-target mixtures in a true experiment carried out in a laboratory. Probes designed by the UPS can be utilized for detecting infectious agents, such as bacteria and viruses, with a decreased chance of cross-hybridizing partners from host transcripts. Therefore, the UPS is very useful when developing a diagnostic kit.

The UPS is suited to creating specific small arrays from selected genes or signatures by subtraction genetic background. However, when input sequences are closely related to each other, only a small fraction of genes will likely have probes according to UPS criteria In the next step, a relatively more sophisticated algorithm will be generated to reduce computation time and accept relative more experimental conditions. Although the UPS can be applied on design arrays in whole genome scale, large datasets (>40 M) are not accepted. Requirements for probe design from a large dataset can be forwarded to the authors for solutions.

## Methods

### System implementation

The UPS was run on two symmetrical multi-processor (SMP) PCs equipped with dual CPUs (Intel Pentium-Xeon 3.4 GHz) and 7 GB of RAM. For efficiency and performance, the LAMP structure was adopted, which is the abbreviation of Linux (Mandrake 2007 operating system)-Apache (version 2.04, web server)-PostgreSQL (version 8.2.4, object-relational database)-PHP (version 5.1.0, html-embedded scripting language), to provide the web access, file upload and download services, mail notification and data storage.

Figure 1 presents the workflow of the UPS. All calculations concerning suffix arrays, constraints for probe selection (GC%, low complexity masking, distance from the 3' end), energy for probe folding and melting temperature in a thermodynamic model and *in silico *hybridization assessment based on blastn (check for sequence identity and maximum overlap amid probe and non-target sequences) [13] were performed on an MS-Window XP-based SMP in Boland Delphi 2006. Several approaches were used to estimate tendency for cross-hybridization, including free energy, sequence identity of probe to all possible templates, and sequence complexity of probes. To avoid probe secondary structure, a Perl program, UNAFold.pl, was integrated into the UPS to calculate ΔG [14].

### Basic criteria for probe selection

#### 1. Probe length

In the UPS, probe length is 30–120 nucleotides, which yields a good compromise between specificity and calculation noise [8].

#### 2. Melting temperature

The probe annealing temperature (Ta) is determined based on melting temperature (Tm). Probe Tm depends on several physiochemical factors and is calculated in the following equation based on Nearest-Neighbor model [15]:

ΔTm = ΔH/(10.8 + ΔS + R × ln(C/4)) - 273.15 + 16.6(log_10_[Salt])

where ΔH is the sum of enthalpy of the nearest neighbors, ΔS is the sum of entropy of the nearest neighbors, C is total molar strand concentration (1 nM in this case), and R is Boltzmann's constant (1.987). The default concentration of [Salt] is in 0.58 M. In the UPS default setting, the GC content of probes is 30–70%.

#### 3. Sequence complexity

Low probe complexity reduces discriminatory power for the sequence content. Any five or more continual nucleotides (AAAAA, TTTTT, CCCCC, or GGGGG) are excluded. Continuous di-nucleotide/tri-nucleotide repeats, such as 'ATATAT' and 'ATGATGATG', are also eliminated.

#### 4. Computation of secondary structure formation

The Perl program UNAFold.pl is integrated into the UPS to generate the ΔG value [14]. Oligos with a negative minimum folding energy at its Tm and 0.58 M salt concentration are apt to form secondary structures.

#### 5. Continuous stretch and identity between probe and no-target template

Li *et al*. investigated the relationships between hybridization signals and sequence identity, continuous stretch, binding free energy and mismatch position [16]. By combining multiple criteria, they suggested liberal cut-offs can be employed for each criterion. This work used their experimentally established criteria to exclude unsuitable oligonucleotides: 1) identity, ≦85%; 2) continuous stretch of ≧17; and, 3) free energy <-35 kcal/mol (depending on probe length) of probe-non-target duplex. The Nearest-Neighbor model and blastn were applied to identify probe candidates for each input sequence with a low likelihood of cross-hybridization.

## Competing interests

The authors declare that they have no competing interests.

## Authors' contributions

All authors have contributed together towards this goal. CL conceived of this study, integrated all the information, sketched the succinct web design and drafted the manuscript. SC participated in manuscript preparation, discussions and provided many useful suggestions for web interface and presentation. CL (Chen-Ren Lo) carried out a major part of this work including implementing algorithm for calculation and automatic update mechanism. MT (Ming-Chi Tsai) devoted a lot of time in implement whole infrastructure including the design on schema of database, web GUI for dynamic network, robustness and security of Linux-Apache-Mysql-PHP platform. CAH supervised and directed the development process of the whole project, conforming to ethical principles, critical examination and manuscript preparation. All authors have read and approved the final manuscript.
